# Quantitative Analysis of Mucin Expression Using Combined Alcian Blue-Periodic Acid Schiff (AB-PAS) Stain and Combined High Iron Diamine-Alcian Blue (HID-AB) Stain and the Correlation With Histomorphological Score in Chronic Calculous Cholecystitis

**DOI:** 10.7759/cureus.32033

**Published:** 2022-11-29

**Authors:** Karthikeyan Vadivazhagan, Kalaivani Amitkumar, Muthu Sudalaimuthu

**Affiliations:** 1 Pathology, Mahatma Gandhi Medical College and Research Institute, Pondicherry, IND; 2 Pathology, SRM Medical College Hospital And Research Centre, Chengalpattu, IND; 3 Pathology, SRM Medical College Hospital And Research Centre, Chennai, IND

**Keywords:** histochemical stain, cholecystectomy, gall stones, mucin, cholecystitis

## Abstract

Introduction

Chronic calculous cholecystitis is the most common and important contributing factor for cholecystectomy across the country, with a prevalence of 2-29%. Cholesterol supersaturated bile plays a major role in stone formation. It is very essential to identify the pathogenesis of stone formation in order to prevent its formation. This study is aimed to evaluate histomorphological features of chronic calculous cholecystitis and to quantitatively evaluate alteration in mucin expression using the combined Alcian blue-periodic acid Schiff (AB-PAS) stain and the combined high iron diamine Alcian blue (HID-AB) stain, to correlate with each other and also with biochemical features of gall stones.

Methods

A cross-sectional study of 64 chronic calculous cholecystitis were taken for histomorphological assessment and grading using Hematoxylin and Eosin (H&E) stain and Masson's trichrome stains. Expression of the type of mucins was analyzed using histochemical stains by a standardized scoring system.

Results

A significant positive correlation was observed between an increase in grades of inflammation and fibrosis with an increase in the quantity of sialomucin and neutral mucin in the deep layers of epithelium, and a significant negative correlation was observed between an increase in grades with a decrease in acidic mucin and sulfomucin of both superficial and deep epithelium except sulfomucin in fibrosis. No significant correlation was obtained with muscle thickness, adipose tissue deposition, and epithelial hyperplasia. A higher frequency of mixed-type stones was associated with severe inflammation.

Conclusion

Inflammation and fibrosis were strongly correlated with quantitative alteration and reversal of mucin composition in chronic cholecystitis; hence we conclude that these features play a significant role in the pathogenesis of stone formation. Using Combined AB(2.5pH)-PAS stain and Combined HID-AB(2.5 pH) stain to detect mucin hypersecretion and composition of altered mucin is relatively accurate and cost-effective rather than performing costly immunohistochemical (IHC) markers.

## Introduction

Cholecystitis is one of the leading contributing factors for hospital admission, morbidity, and cholecystectomy [1]. Among the world's population, the incidence of cholecystitis is 10 to 20%. In India, the overall prevalence is 2 to 29%, out of which North India shows seven times more prevalence than South India [2]. The most important contributing factors for gallstone formation include cholesterol supersaturated bile which is lithogenic, along with alteration of gallbladder mucin, calcium, and gallbladder dysfunction related to motility, emptying, contractility, and enterohepatic circulation. For making bile lithogenic, the biliary calcium, which is one of the contributing factors, will decrease the cholesterol solubility in bile and also become one of the contents in the composition of gallstones [3].

The role and contribution of gallbladder mucin in the pathogenesis of cholelithiasis is studied extensively, and many studies showed that during gallstone development, mucin undergoes hypersecretion and forms nuclei and structural parts of calculi. Another contributing factor is prostaglandin which triggers gallbladder mucosa to secrete mucin. The major mucin content present in the normal gallbladder epithelium is predominantly sulfated mucin with a minimal quantity of sialomucin and neutral mucin. The proportion of this mucin composition gets altered in chronic calculous cholecystitis. With an epithelial metaplastic change in the epithelium, the sulfomucin decreases with the rise in sialomucin. Changes in the mucin expression in the gallbladder play a vital role in the pathogenesis of gallstone disease. Hence, it is essential to identify the pathogenesis of stone formation with respect to mucin alteration in order to prevent its formation [3-5].

The aim of this study is to assess histomorphological features as well as histochemical mucin expression using combined Alcian blue-periodic acid Schiff 2.5pH (AB-PAS) stain and combined high iron diamine-Alcian blue with 2.5pH (HID-AB) stain and correlation with each other and also with biochemical features of calculi in chronic calculous cholecystitis. The combined stain of HID-AB stain is not used routinely in laboratories; hence we also intend to observe the usefulness of this rare stain in identifying mucin alteration.

## Materials and methods

The study was initiated following approval from the Institutional Ethics and Scientific Committee, and written informed consent was obtained from the patients. A cross-sectional single-institute study was conducted in the Sri Ramaswamy Memorial (SRM) Medical College Hospital and Research Centre in Tamilnadu. The sample size was 64 cases of cholecystectomy specimens. Fifteen cases were retrospective cases, and 49 cases were prospective.

Cholecystectomy specimens received in the histopathology section of the pathology department with a clinical diagnosis of chronic calculous cholecystitis were included during the study period of august 2019 to august 2021.

Chronic cholecystitis specimens without gallstones, specimens received in fragments without orientation or integrity, gallbladder with malignancy, and autolyzed or poorly fixed specimens were excluded from the study.

Clinical findings, including age and sex details, were obtained. For prospective cases, open or laparoscopic cholecystectomy specimens along with calculi were obtained from the department of surgery in a container filled with 10% buffered formalin. For analyzing retrospective cases, the specimens, stones, histopathological reports, paraffin blocks, and slides were extracted from the department archives. In each and every case the standard grossing procedure of gallbladder specimen was followed, and appropriate samples from the fundus, body, and neck were taken.

Analysis of histomorphological features

For histomorphological analysis, sections were stained using routine Hematoxylin and Eosin (H&E) stain for evaluating features such as inflammation, fibrosis, muscle thickness, adipose tissue deposition, and hyperplastic epithelium. Sections with proper mucosal epithelium were identified, and additional sections from those blocks were cut to assess fibrosis and muscle thickness using Masson's trichrome histochemical stain. For grading of the above-mentioned features, a modified scoring system was used (Tables 1, 2). 

**Table 1:** Grading and scoring of inflammation and fibrosis

Grades of inflammation	Microscopic findings
Mild (1+)	Diffuse mononuclear infiltrate of less than or equal to 10 cells /HPF in any layer.
Moderate (2+)	Diffuse mononuclear infiltrate between 11 -30 cells/HPF.
Severe (3+)	Diffuse mononuclear infiltrate >31 cells/HPF or follicular formation.
Grades of fibrosis	Microscopic findings
Mild (1+)	Unequal deposition of collagen in less than or equal to 20 % of the material.
Moderate (2+)	Unequal deposition of collagen in 21-70% of the material.
Severe (3+)	Unequal deposition of collagen or lamellar fibroplasias in more than or equal 71% of the material.

**Table 2:** Grading and scoring of muscle thickness, adipose tissue deposition, and mucin histochemistry HID-AB - high iron diamine Alcian blue, AB-PAS - Alcian blue-periodic acid Schiff

Grading of muscle thickness (using Masson's trichrome stain)	
Grades	Microscopic findings
Mild (1+)	<1/3 of the entire wall thickness
Moderate (2+)	1/3 -2/3 of the entire wall
Severe (3+)	>2/3 of the entire wall
Adipose tissue deposition	
Grades	Microscopic findings
Mild (1+)	<10% of the material.
Moderate (2+)	11-60% of the material.
Severe (3+)	>60% of the material.
Scoring system for mucin histochemistry AB-PAS and HID-AB stain	
Percentage of positive cells/field (under 10X magnification)	Score
75 to 100% cells	5+
50 to 75% cells	4+
25 to 50% cells	3+
5 to 25% cells	2+
0 to 5% cells	1+

Analysis of mucin histochemistry

For analyzing mucin histochemical changes, two special stains were used, namely combined Alcian blue (2.5 pH) and periodic acid Schiff (AB-PAS) and combined stain of high iron diamine and Alcian blue (2.5 pH) (HID-AB). Standardized methodology for both stains is given below:.

Combined Stain of AB-PAS

Initially, deparaffinization and hydration of tissue section were made with the help of xylene changes I, II, and III, followed by processing with decreasing grades of alcohol concentration. Sections were kept in running tap water; each change lasted for five minutes. Alcian blue solution was added and kept aside for 30 minutes, followed by five minutes of running tap water and distilled water wash. For the oxidation step, the periodic acid reagent was added and kept undisturbed for five to 10 minutes, followed by five minutes of running tap water and distilled water wash. Schiff reagent was poured until it fully covered the section and kept for 20 minutes, followed by 10 minutes of running tap water wash. Counterstained the section with Hematoxylin, followed by blueing with the help of running tap water wash for five minutes. Finally, five minutes of tap water wash was done. Rapid dehydration with increasing grades of alcohol concentration followed by clearing with xylene was done. At the end, sections were mounted.

Combined Stain of HID-AB

Initially, deparaffinization and hydration were completed similarly to the previously mentioned protocol. Sections were fully immersed with high iron diamine solution for 18-24 hours, and then washed in running tap water for five to 10 minutes. Then slides were stained with Alcian blue solution for 30 minutes, followed by running tap water wash. Counterstained using 0.5% neutral red solution for one to two minutes. Then water wash, rapid dehydration, and clearing steps were applied, as previously mentioned. Finally, they were mounted with a mounting medium.

Mucin expression of the superficial and deep lining epithelium of chronic calculous cholecystitis was assessed using a detailed scoring system mentioned in Table 2.

Detection of type of gallstones using biochemical stone analysis

Stones were washed and dried thoroughly, then crushed into powder. Biochemical tests were performed as per standard protocol to detect components such as cholesterol, bilirubin, both direct and indirect, bile salt, oxalate, and calcium phosphate. For assessing liver function derangement, liver function test values were collected from the biochemistry department.

Statistical analysis

Distribution of age, sex, and various histomorphological parameters were presented using percentage (%) and frequency. Correlation between histomorphological and mucin histochemical features was done using Chi-square and Pearson correlation methods. Percentage and frequency were used for identifying the incidence of inflammatory grades with types of calculi. Association between gall stones and liver function test was analyzed using likelihood ratio tests of multinomial logistic regression method. SPSS version 21.0 (IBM Inc., Armonk, New York) was the software with which statistical analysis was done.

## Results

Out of 64 cholecystitis cases, the most commonly affected age group was 31 to 40 years (38%), followed by 21 to 30 years, with a female preponderance of 51 cases (80%). Thirty-eight out of 64 cases (59%) of chronic calculous cholecystitis showed mixed stones, and 42/64 cases (66%) showed the presence of multiple stones.

Distribution of histomorphological features

**Figure 1:**
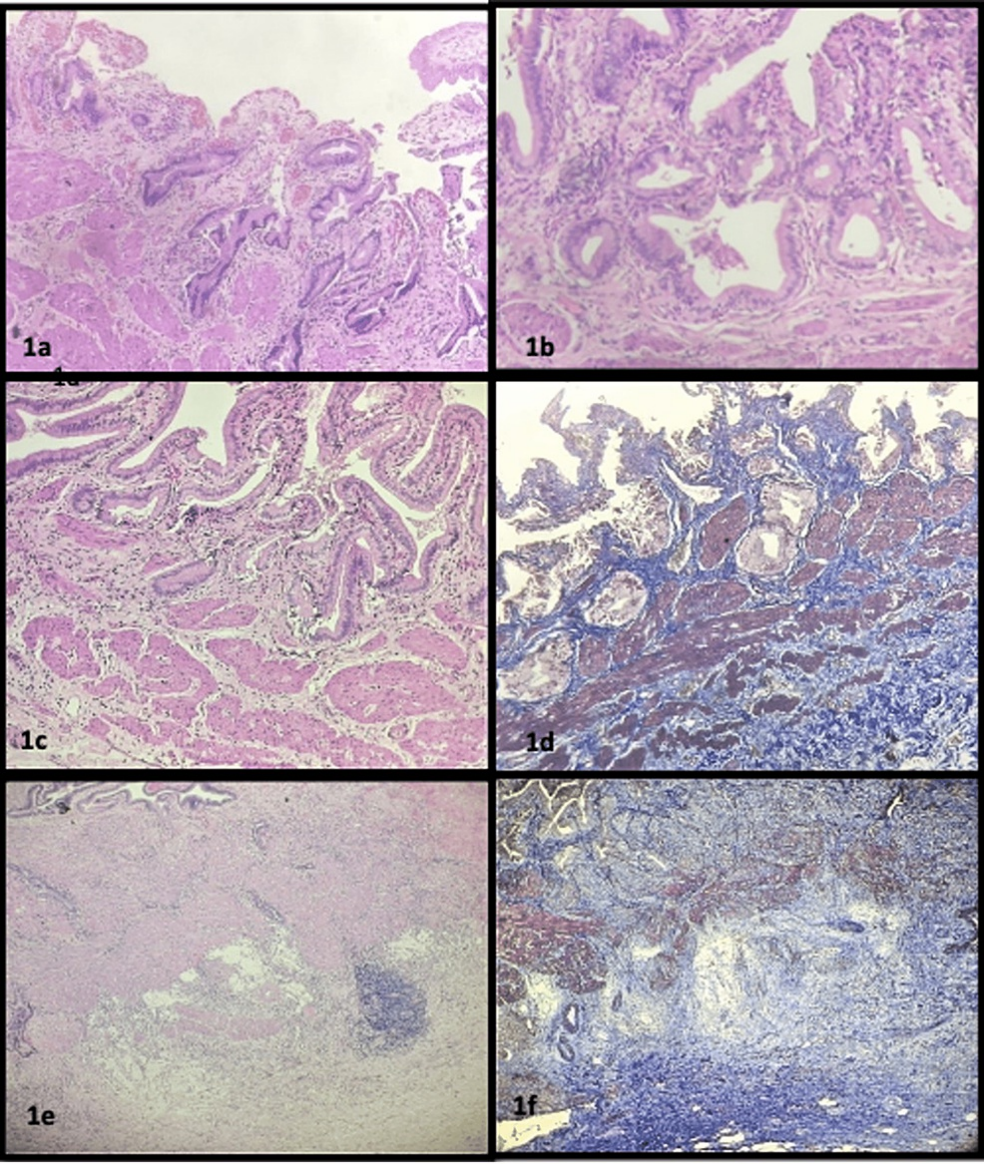
Chronic calculous cholecystitis with various grades of inflammation and fibrosis Moderate (a) inflammation (2+) and severe (b) inflammation (3+) with focal lymphoid aggregates (H&E,10X), Moderate fibrosis (2+) in H&E (c) and Masson's trichrome stain (d) (10X), severe fibrosis (3+) using H&E (e) and Masson's trichrome stain (f) (10X)

Out of 64 cases, 15 cases (23%) showed mild inflammation (grade I), 24 cases (38%) showed moderate inflammation (grade II), and 25 cases (39%) showed severe inflammation (grade III) (Figure 1a, 1b). Mild fibrosis was observed in 26 cases (40.6%) considered as grade I, 30 cases (46.9%) showed moderate fibrosis (grade II), and eight cases (12.5%) showed severe fibrosis (grade III), analyzed and verified using Masson's trichrome stain shown in Figure 1c, 1d, 1e, and 1f. On analysis of muscle thickness, most of the cases (46,71.8%) showed features of grade I ( mild), 14 cases (21.8%) were found to be grade II (moderate), and only four cases (6.3%) fell under grade III (severe). On observing adipose tissue deposition, most of the cases (38, 59.4%) fell under grade I (mild), 23 cases (35.9%) were in grade II (moderate), and only three cases (4.7%) were identified as grade III (severe). Twelve cases (18.7%) showed focal epithelial hyperplasia, 10 cases (15.6%) showed diffuse hyperplasia, and 42 cases (65.6%) showed an absence of significant hyperplastic epithelium. 

Mucin histochemical analysis

**Figure 2:**
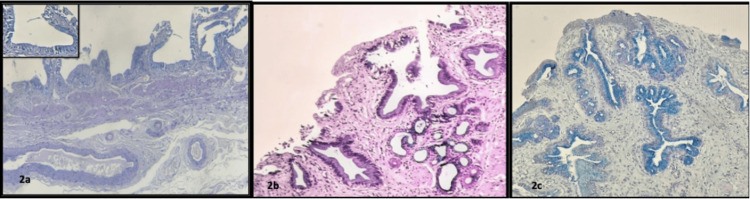
AB-PAS stained sections showing varying scores of mucin a) score one - less than 5% of mucin (acidic) containing cells taken up by Alcian blue (inset highlighted AB positive focus); b) score three - 25 to 50% of acidic and neutral mucin-containing cells taken up by both Alcian blue and PAS stain; c) score four - 50 to 75% of mucin (acidic) containing cells (AB-PAS, 10X) AB - Alcian blue, PAS - periodic acid Schiff

**Figure 3:**
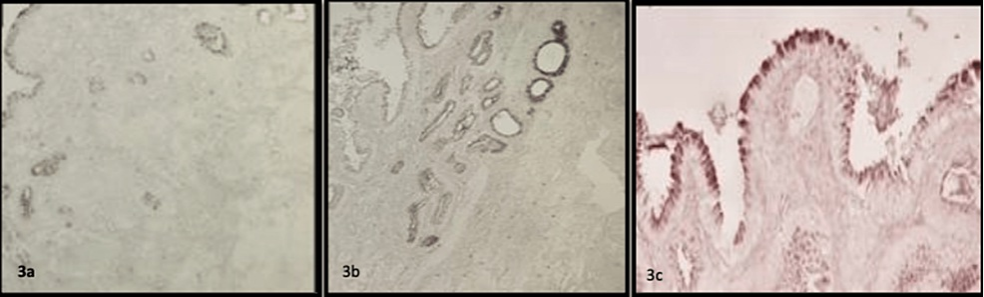
HID-AB stained sections showing varying scores of mucin a) score two - 5 to 25% of mucin (sulfomucin) containing cells; b) score three - 25 to 50% of mucin (sulfomucin) containing cells; c) score five - 75 to 100% of mucin (sulfomucin) containing cells (inset highlights HID positive focus) (HID-Ab stain 10X) AB - Alcian blue, HID - high iron diamine

Mucin expression of superficial and deep lining epithelium of chronic calculous cholecystitis was assessed using a detailed scoring system [6]. Sections from all specimens stained with both stains were quantitatively scored. AB-PAS-stained sections showing varying scores of mucins ranging from scores one to five are shown in Figure 2, and HID-AB stained sections showing varying scores of mucin, such as score one to score five, are depicted in Figure 3.

Mucin expression correlated to grades of inflammation

Acidic mucin expression of superficial and deep epithelium showed a decrease in the mean score from 3.65 to 1.66 and 3.79 to 2.08 with respect to the increase in grades of inflammation. Neutral mucin expression of superficial and deep epithelium showed an increase in the mean score among acidic mucin, sulfomucin expression of superficial and deep epithelium showed a decrease in the mean score, and sialomucin expression showed an increase in the mean score with respect to an increase in grades of inflammation. A statistically significant negative correlation was obtained between increasing grades of inflammation and decreasing expression of acidic mucin and sulfomucin. A statistically significant positive correlation was obtained between increasing grades of inflammation and an increased expression of sialomucin and neutral mucin; however, sialomucin and neutral mucin in superficial epithelium did not show statistical significance (Table 3).

**Table 3:** Correlation of mucin expression with grades of inflammation and fibrosis in superficial and deep epithelium using AB-PAS stain and HID-AB stain Correlation between histomorphological and mucin histochemical features was done using Chi-square and Pearson correlation methods AB-PAS - Alcian blue-periodic acid Schiff, HID-AB - high iron diamine-Alclan blue

Parameters	Grade (cases)	Alcian blue (AB)				Periodic acid Schiff (PAS)				High iron diamine (HID)				Alcian blue (AB)			
		Superficial		Deep		Superficial		Deep		Superficial		Deep		Superficial		Deep	
		Mean	p-value	Mean	p-value	Mean	p-value	Mean	p-value	mean	p-value	mean	p-value	mean	p-value	mean	p-value
Inflammation	Mild (38)	3.65	0.00	3.79	0.00	0.61	0.28	0.57	0.04	3.92	0.00	3.98	0.00	0.08	0.117	0.03	0.004
	Moderate (37)	2.58		2.77		0.98		0.94		2.86		3.02		0.09		0.1	
	Severe (29)	1.66		2.08		1.09		1.3		1.96		2.42		0.17		0.24	
Fibrosis	Mild (39)	2.86	0.005	3.24	0.00	0.65	0.07	0.71	0.01	2.97	0.096	3.23	0.12	0.09	0.246	0.07	0.02
	Moderate (43)	2.38		2.57		1.03		1.07		2.79		2.97		0.13		0.17	
	Severe (22)	1.36		1.82		1.53		1.62		1.93		2.45		0.18		0.23	

Mucin expression related to grades of fibrosis

Acidic mucin expression of superficial and deep epithelium showed a decrease in mean score from 2.86 to 1.36 and 3.24 to 1.82 with respect to the increase in grades of fibrosis. Neutral mucin expression of superficial and deep epithelium showed an increase in the mean score, sulfomucin expression showed a decrease in the mean score, and sialomucin expression showed an increase in mean score from 0.09 to 0.18 and 0.07 to 0.23 with respect to an increase in grades of fibrosis. A statistically significant negative correlation was obtained between increasing grades of fibrosis and decreasing expression of acidic mucin of superficial and deep epithelium. However, sulfomucin in superficial and deep epithelium did not show significance. A statistically significant positive correlation was obtained between increasing grades of fibrosis and increasing expression of sialomucin and neutral mucin; however, sialomucin and neutral mucin in superficial epithelium did not show statistical significance (Table 3)

Correlation of mucin expression with muscle thickness

In the current study, acidic mucin expression of superficial and deep epithelium showed a decrease in mean score from 2.52 to 2.2 with respect to an increase in muscle thickness, but the mean score of moderate and severe grades of muscle thickness did not show a significant difference, as shown in Table 4. The neutral mucin expression of superficial and deep epithelium showed an increase in the mean score. The sulfomucin expression of superficial and deep epithelium showed a decrease in the mean score. The sialomucin expression of superficial and deep epithelium showed no significant variation but only a minimal increase in the mean score. The correlation between the increase in grades of muscle thickness and expression of mucin in both epitheliums was not statistically significant. 

**Table 4:** Analysis of acidic, neutral, sulfomucin, and sialomucin expression with respect to muscle thickness Correlation between histomorphological and mucin histochemical features was done using Chi-square and Pearson correlation methods AB - Alcian blue, PAS - periodic acid Schiff, HID - high iron diamine

Thickness of muscle layer	AB (Acidic mucin)				PAS (Neutral mucin)				HID (Sulfomucin)					AB (Sialomucin)				
	Superficial		Deep		Superficial		Deep		Superficial		Deep			Superficial			Deep	
	Mean	p-value	Mean	p-value	Mean	p-value	Mean	p-value	Mean	p-value	Mean	p-value	Mean		p-value	Mean		p-value
Mild (46)	2.52	0.38	2.86	0.25	0.89	0.64	0.96	0.86	2.85	0.35	3.12	0.24	0.11		0.81	0.13		0.82
Moderate (14)	2.2		2.4		1		1		2.56		2.91		0.15			0.13		
Severe (4)	2.2		2.4		1.43		1.36		2.23		1.86		0.13			0.23		

Correlation of mucin expression with adipose tissue deposition

Acidic mucin expression of superficial and deep epithelium showed a decrease in mean score from 2.76 to 1.46 and 2.93 to 1.93 when compared to the increase in adipose tissue deposition. Neutral mucin expression of superficial and deep epithelium showed an increase in the mean score, sulfomucin showed a mild decrease in the mean score, and sialomucin showed almost the same mean score. The correlation between the increase in grades of adipose tissue deposition and expression of all types of mucin in both epithelium, except acidic mucin expression of superficial epithelium, was not statistically significant. 

Mucin expression correlated to epithelial hyperplasia

Acidic mucin expression of superficial and deep epithelium in diffuse hyperplasia showed a mean score of 2.7 and 2.7; focal hyperplasia showed a mean score of 2.1 and 2.4, and absence of hyperplasia showed a mean score of 2.5 and 2.9, respectively. The sulfomucin expression of superficial and deep epithelium in diffuse hyperplasia showed the highest mean score of 3.39 and 3.67; focal hyperplasia showed 2.26 and 2.43. The sialomucin expression of superficial and deep epithelium in diffuse hyperplasia showed 0.17 and 0.2. The presence of epithelial hyperplasia did not show a significant correlation with any mucin expression. 

Frequency of number, type of gallstones, and correlation with grades of inflammation

**Figure 4:**
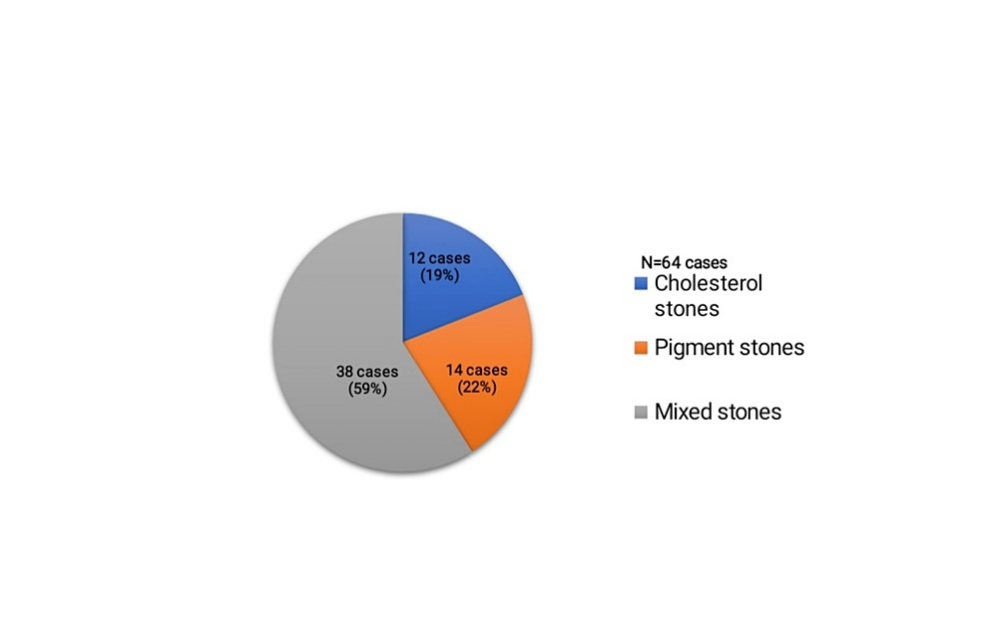
Distribution of gallstones among the study population Stones were typed based on biochemical analysis

Among 64 cases, 38 cases were mixed stones, 14 cases were pigment stones, and 12 cases were cholesterol stones (Figure 4)

Forty-two cases showed multiple stones, and 22 cases were found to have a single stone. On assessing the frequency of gallstone types with respect to grades of inflammation, cases with mild inflammation (15 cases) showed equal distribution of all three types of stones, whereas cases with moderate (24 cases) and severe inflammation (25 cases) showed a predominance of mixed stones (Table 5).

**Table 5:** Frequency of gallstone types with respect to grades of inflammation

Grades of inflammation (N=64)	Mixed stones	Pigment stones	Cholesterol stones
Mild (15 cases)	5 (34%)	5 (33%)	5 (33%)
Moderate (24 cases)	15 (62%)	5 (21%)	4 (17%)
Severe (25 cases)	18 (72%)	4 (16%)	3 (12%)

## Discussion

In the present study, we quantitatively analyzed and correlated three aspects namely histomorphological features, alteration of mucin expression, and biochemical characteristics of calculi in chronic calculous cholecystitis specimens. Most of the previous studies had combined any one or two aspects only. On a detailed literature search, only two previous studies were observed where all three aspects were correlated, as done in this study. Mucin, a glycoprotein, forms a part of the structural component of gallstones and also plays a major role in the pathogenesis of stone formation. Hence, it was important and necessary to identify the quantity of alteration in mucin expression in the gallbladder mucosa of chronic calculous cholecystitis specimens and their correlation with histomorphological features, which is expected to help in narrowing down to the specific type of mucin formation and progression of gallstone disease. 

In the present study, the most common age group affected was 31 to 40 years which is in concordance with other studies done by Anupama et al., Thamilselvi et al., and Mathur et al. [6-8]. This age group predilection might be attributed to dyslipoproteinemia; as age progresses, there is an increase in the release of cholesterol into the bile associated with a decrease in bile synthesis. In the current study, the male-to-female ratio was 1:4, which is in concordance with Goyal et al., who observed a 1:4.2 ratio in their study. Most of the cases fell under the moderate and severe degree of inflammation, 24 (38%) and 25 (39%) cases, respectively, which were similar to the study done by Anupama et al., where inflammation in cholecystitis was due to obstruction by calculi, which in turn leads to mucosal injury. Prolonged inflammation reveals a predominant B lymphocyte population, and when neutrophilic activity accompanies the mild to moderate chronic inflammation, it also exhibits T lymphocytes which are limited to affected areas [6,9].

Based on the degree of fibrosis observed and compared using both H&E stain and Masson's trichrome stain, a predominantly mild and moderate degree of fibrosis was observed. The results were comparable with studies done by Barcia and Anupama et al., where they found that the frequency of mild and severe degrees of fibrosis was almost closer to each other [5,6]. Fibrosis could be due to incomplete healing of an acute injury, which leads to collagen deposition initially in the disordered pattern followed by rare orderly/lamellated arrangement in long-standing cases of chronic cholecystitis. A mild (grade I) increase in the thickness of muscle was observed in many cases; these were also inferred by some studies, such as Zhou et al., who found that 150/ 378 (40%) cases, and Barcia, who found that 55/100 (55%) cases showed a mild increase in thickness of muscle layer [4,5]. This frequently occurs due to a rise in intraluminal pressure caused by gallstone obstruction. This led to the formation of Rokitansky-Aschoff sinuses and muscular hypertrophy [10].

Mild deposition of adipose tissue was observed in 38 (59.4%), which showed concordance with the study conducted by Zhou et al. and Barcia [4,5]. They also observed that a mild amount of adipose tissue deposition was present in stage II, and moderate to severe deposition was observed in stage III of chronic cholecystitis. In this study, 34.3% showed the focal type of epithelial hyperplasia, which was slightly more predominate than the diffuse type. These findings are similar to studies done by Baidya et al. and Singh et al. Epithelial hyperplasia could be a reactive response to repeated irritation and regeneration following exposure of mucosal lining to concentrated bile [10-13].

We attempted to correlate the mucin expression with all the histomorphological parameters already explained and observed a significant positive correlation between an increase in grades of severity of inflammation and fibrosis, with an increase in the quantity of sialomucin and neutral mucin in the deep layers of the epithelium. A significant negative correlation was observed between an increase in grades of severity with a decrease in acidic mucin and sulfomucin of both superficial and deep epithelium, except sulfomucin in fibrosis. These findings were identical to the study conducted by Srivastava et al. and Anupamaet al. [1,6]. Pani et al. found that a major amount of mucin in cholelithiasis and cholecystitis was sulfomucin and sialomucin, respectively. Similar to intestinal epithelium, the gallbladder also showed increased mucin secretion for stone formation and acceleration which leads to concomitant exhaustion of mucin content within the lining epithelium [14,15]. Finally, in this study, we found that inflammation causes mucin alteration, which is exactly opposite to normal gallbladder mucin. Therefore cases of severe inflammation show a maximum increase in sialomucin and neutral mucin. Mucin alteration in severe fibrosis was similar to severe inflammation.

We found that when there was an increase in the thickness of muscle layer and deposition of adipose tissue, there were changes in mucin expression similar to inflammation and fibrosis, except sialomucin expression, which remains unchanged with the increase in deposition of adipose tissue. In cases with diffuse hyperplastic epithelial changes, an increase in sulfomucin, a mild increase in sialomucin, and a decrease in neutral mucin were noted. To our knowledge, very few studies studied the above correlation. Mucin alteration in cases with severe smooth muscle hypertrophy was similar to severe inflammation. The above changes in mucin with respect to hyperplasia reveal that mucin secretion in mucosal hyperplasia was due to activation of PGE2 following exposure of mucosa to cholesterol-rich lithogenic bile prior to stone formation. Immunohistochemical (IHC) markers MUC3, MUC5AC, MUC5B, and MUC6 were expressed in gallbladder epithelium, and MUC5AC and MUC5B were also identified in the bile. A significant level of MUC5AC expression was associated with inflammation and pigmented brown gallstones. A statistically significant positive correlation was obtained between increasing grades of inflammation and fibrosis along with an increased expression of different types of mucin using the combined histochemical stains in our study, hence application of combined histochemical stains to detect altered mucin in chronic cholecystitis specimens is cost-effective rather than performing large panel of costly IHC markers [16,17].

Forty-two cases (66%) showed the presence of multiple stones, which was found to be identical to previous studies [6,9]. This implied that multiple stones more commonly caused symptomatic calculous cholecystitis than a single stone [12]. Following biochemical analysis, we found that mixed stones were the most frequent type of gallstones (38 cases, 59%), and the results were identical to studies done in India [2,6]. This gallstone frequency could be attributed to ethnicity and regional variation in dietary habits and lifestyle changes. A diet containing low fibers, increased refined carbohydrates, high cholesterol, and prolonged parenteral nutrition can lead to the supersaturation of bile with cholesterol. We found a higher frequency of mixed-type stones in severe inflammation, followed by pigment stones and cholesterol stones, whereas Anupama et al. in their study found that the frequency of pigment stones was more common than cholesterol stones in severe inflammation [6]. Vilkin et al. stated that a higher frequency of brown pigment stones with higher grades of inflammation due to bacterial infection, which hydrolyses phospholipid and bilirubin conjugates [17]. Pillai et al. explained that 61% of the cases showed mixed multiple stones, which was in concordance with the present study [18]. Tyagi et al. also showed similar findings [19].

## Conclusions

The most common age group affected was 31 to 40 years, with a 1:4 male-to-female ratio. Most of the cases showed a severe to moderate degree of inflammation, moderate fibrosis, mild muscular hypertrophy, mild adipose tissue deposition, and focal epithelial hyperplasia. Inflammation and fibrosis were strongly correlated with quantitative alteration and reversal of mucin composition in chronic cholecystitis; hence we conclude that all these features play a significant role in the pathogenesis of stone formation.

Using combined AB(2.5pH)-PAS stain and combined HID-AB(2.5 pH) stain to detect mucin hypersecretion and composition of altered mucin in chronic cholecystitis specimens is relatively accurate and cost-effective rather than performing costly IHC markers. There was no statistically significant correlation between mucin expression and grades of muscle thickness, adipose tissue deposition, and hyperplastic epithelium. Most of the cases showed mixed and multiple stones, which are frequently observed in severe/grade III inflammation than pigment and cholesterol stones. This difference in distribution may be due to regional variations in diets and lifestyles. 

## References

[1] Dhamodharan V, Srivastava V (2017). Study of mucin histochemistry in chronic calculous cholecystitis. J Dent Med Sci.

[2] EzhilArasi N, Aruna L, Bushra AB (2015). Clinicopathological study of chronic calculous cholecystitis with chemical analysis of gallstones. Int J Res Health Sci.

[3] Zaki M, Al-Refeidi A (2009). Histological changes in the human gallbladder epithelium associated with gallstones. Oman Med J.

[4] Zhou D, Guan WB, Wang JD, Zhang Y, Gong W, Quan ZW (2013). A comparative study of clinicopathological features between chronic cholecystitis patients with and without Helicobacter pylori infection in gallbladder mucosa. PLoS One.

[5] Barcia JJ (2003). Histologic analysis of chronic inflammatory patterns in the gallbladder: diagnostic criteria for reporting cholecystitis. Ann Diagn Pathol.

[6] Anupama P, Menon Nirmala V, Janaky R, Mohammed IL, Alingal MB, Sideeque NA (2014). A histopathological and histochemical study of cholecystitis. Int J Hepatobiliary Pancreat Dis.

[7] Thamil Selvi RR, Sinha P, Subramanian PM, Konapur PG, Prabha CV (2011). Clinico-pathological study of cholecystitis with special reference to analysis of cholelithiasis. Int J Basic Med Sci.

[8] Mathur SK, Duhan A, Singh S (2012). Correlation of gallstone characteristics with mucosal changes in gall bladder. Trop Gastroenterol.

[9] Goyal S, Singla S, Duhan A (2014). Correlation between gallstones characteristics and gallbladder mucosal changes: a retrospective study of 313 patients. Clin Cancer Investig J.

[10] Baidya R, Sigdel B, Baidya N (2012). Histopathological changes in gallbladder mucosa associated with cholelithiasis. J Pathol Nep.

[11] Singh A, Singh G, Kaur K, Goyal G, Saini G, Sharma D (2019). Histopathological changes in gallbladder mucosa associated with cholelithiasis: a prospective study. Niger J Surg.

[12] Madrid JF, Ballesta J, Galera T, Castells MT, Pérez-Tomás R (1989). Histochemistry of glycoconjugates in the gallbladder epithelium of ten animal species. Histochemistry.

[13] Ganesh IM, Subramani D, Halagowder D (2007). Mucin glycoarray in gastric and gallbladder epithelia. J Carcinog.

[14] Pani DJ (2013). Histological changes in human gallbladder in pathological condition including cholecystitis and cholelithiasis an analytical study. IOSR J Pharm.

[15] Gupta SC, Misra V, Singh PA, Roy A, Misra SP, Gupta AK (2000). Gall stones and carcinoma gall bladder. Indian J Pathol Microbiol.

[16] Haldar B, Chowdhuryan R, Sarkar M, Talukdar M, Mitra S (2018). Histochemical study of inflammatory lesions of gall bladder with reference to metaplastic conditions. J Clin Diagn Res.

[17] Vilkin A, Nudelman I, Morgenstern S (2007). Gallbladder inflammation is associated with increase in mucin expression and pigmented stone formation. Dig Dis Sci.

[18] Pillai V, Sreekantan R, Chisthi MM (2017). Gall bladder stones and the associated histopathology-a tertiary care center study. Int J Res Med Sci.

[19] Tyagi SP, Tyagi N, Maheshwari V, Ashraf SM, Sahoo P (1992). Morphological changes in diseased gall bladder: a study of 415 cholecystectomies at Aligarh. J Indian Med Assoc.

